# The C-Terminal Domain of the Novel Essential Protein Gcp Is Critical for Interaction with Another Essential Protein YeaZ of *Staphylococcus aureus*

**DOI:** 10.1371/journal.pone.0020163

**Published:** 2011-05-19

**Authors:** Ting Lei, Xudong Liang, Junshu Yang, Meiying Yan, Li Zheng, Bruce Walcheck, Yinduo Ji

**Affiliations:** Department of Veterinary Biomedical Sciences, College of Veterinary Medicine, University of Minnesota, St. Paul, Minnesota, United States of America; University of Edinburgh, United Kingdom

## Abstract

Previous studies have demonstrated that the novel protein Gcp is essential for the viability of various bacterial species including *Staphylococcus aureus*; however, the reason why it is required for bacterial growth remains unclear. In order to explore the potential mechanisms of this essentiality, we performed RT-PCR analysis and revealed that the *gcp* gene (*sa1854*) was co-transcribed with *sa1855*, *yeaZ* (*sa1856*) and *sa1857* genes, indicating these genes are located in the same operon. Furthermore, we demonstrated that Gcp interacts with YeaZ using a yeast two-hybrid (Y2H) system and *in vitro* pull down assays. To characterize the Gcp-YeaZ interaction, we performed alanine scanning mutagenesis on the residues of C-terminal segment of Gcp. We found that the mutations of the C-terminal Y317-F322 region abolished the interaction of Gcp and YeaZ, and the mutations of the D324-N329 and S332-Y336 regions alleviated Gcp binding to YeaZ. More importantly, we demonstrated that these key regions of Gcp are also necessary for the bacterial survival since these mutated Gcp could not complement the depletion of endogenous Gcp. Taken together, our data suggest that the interaction of Gcp and YeaZ may contribute to the essentiality of Gcp for *S. aureus* survival. Our findings provide new insights into the potential mechanisms and biological functions of this novel essential protein.

## Introduction

The prevalence of methicillin-resistant *Staphylococcus aureus* (MRSA) and vancomycin-resistant *S. aureus* (VRSA) has caused serious public health concerns worldwide [1], [2]. The limited options of antibiotics for the treatment of infections associated with MRSA and/or VRSA highlight an urgent need for the development of novel potent antimicrobial agents. Bacterial essential proteins are potential targets for the development of new classes of antibiotics [3], [4]; however, the biological function of many essential proteins is still unclear. The characterization and validation of functional unknown essential proteins are therefore of great importance to assess their suitability as targets for the development of the novel antibiotics.

Our previous studies have indicated that a novel essential protein Gcp is a potential target for the development of new classes of antibacterial agent against MRSA and/or VRSA [5], [6]. Gcp is homologous to the Gcp first identified in *Mannheimia haemolytica* (formerly *Pasteurella haemolytica*) and specifically cleaves *O*-sialoglycosilated protein [7]. Gcp homologs are ubiquitous in all three kingdoms of life, with the exception of the endosymbiotic bacteria *Carsonella ruddii* and *Sulcia muelleri*, which possess highly reduced genomes [8], [9]; however, these Gcp homologs do not exhibit glycoprotease activity. It has been revealed that these Gcp homologs are required for cell viability of many bacterial species examined to date, including *S. aureus* [10], *Streptococcus pneumonia* [10], *Escherichia coli* [11], *Bacillus subtilis* [12], [13], *Francisella novicida* [14], *Pseudomonas aeruginos*a [15], and *Mycoplasm genetalium* [16]. Additionally, the protein is important for eukaryotes, such as *Saccharomyces cerevisiae* [17] and *Arabidopsis thaliana* [18]. We have demonstrated that *S. aureus* Gcp plays an important role in the process of bacterial autolysis, suggesting a potential role in the cell wall biosynthesis pathway [6], however, the reason why Gcp is required for bacterial viability remains elusive.

Structural analysis of Gcp homologs shows that Gcp belongs to the ASKHA (acetate and sugar kinases, HSP70 and actin) superfamily [18]–[22]. A conserved metal binding motif HXEXH is inserted within the HSP70 (heat-shock protein70)-actin-like fold (HALF), suggesting a metal binding ability and an ATP dependent protease activity [18]. The crystal structure analysis of the Gcp *Pyrococcus abyssi* ortholog, Pa-Kae1, has revealed that Pa-Kae1is multifunctional, binding iron and ATP, as well as possessing DNA binding and apurinic endonuclease activity [20], whereas the Gcp homolog in yeast, Kae1 (kinase -associated endopeptidase 1), is a component of the KEOPS/EKC (kinase, endopeptidase and other proteins of small size/endopeptidase-like and kinase associated to transcribed chromatin) complex that are required for telomere maintenance and transcription of essential eukaryotic genes [21], [23], [24]. Collectively, the above data indicate that Gcp homologs may possess different functions among different species.

Recently, it has been reported that in *E. coli*, an essential protein YeaZ binds to YgjD (the Gcp homolog in *E. coli*) and YjeE, which are both essential for *E. coli* growth [25], [26]. YeaZ is a bacterial specific member of the ASKHA superfamily. It is also essential for growth of a variety of bacterial species [3], [10]–[15], such as *S. aureus* [10] and *E. coli* [11]. Crystal structural analyses reveal that the *E. coli* [27], *Samonella typhimurium* and *Thermotoga maritime* YeaZs possess a HALF fold, but lack the metal binding motif and the ATP binding site [28], [29]. This suggests that YeaZ may bind to a nucleotide through an interaction with a small molecule ligand or a partner protein to adopt an active conformation [28]. In addition, *Ec*YeaZ can specifically cleave YgjD [26], but *St*YeaZ does not exhibit such an enzymatic activity [28].

Sequence alignment analysis revealed that *S. aureus* Gcp, YeaZ, and SA1857 proteins are the homologs of YgjD, YeaZ, and YjeE in *E. coli*, respectively [26], however, whether these proteins interact with each other in *S. aureus* is currently unknown. In this study, we utilized the genetic and biochemical approaches and demonstrated that the staphylococcal Gcp binds to YeaZ. Importantly, we have identified the key domains of Gcp that are important for Gcp-YeaZ interaction, as well as critical for Gcp's essentiality for *S. aureus* growth.

## Results

### Both *gcp* and *yeaZ* genes are located in the same operon

**Figure 1:**
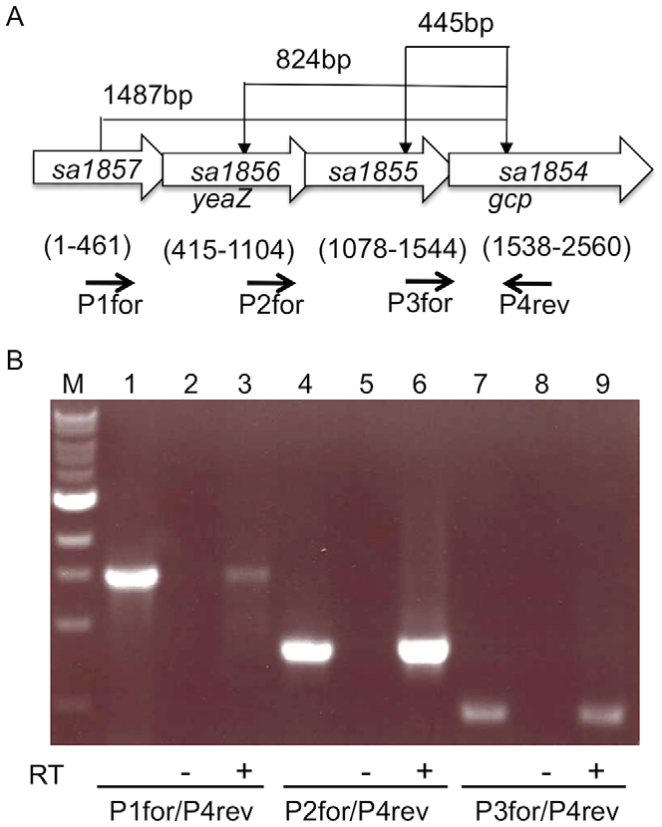
The co-transcription of the *sa1857*, *sa1856* (*yeaZ*), *sa1855*, and *sa1854* (*gcp*) genes. (A) Schematic representation of *sa1857*, *sa1856* (*yeaZ*), *sa1855*, and *sa1854* (*gcp*) genes in *S. aureus*. (B) RT-PCR using different primer pairs. RT (−), total RNA of *S. aureus* used as the template; RT (+), cDNA used as the template; positive controls, the genomic DNA used as the template (lane 1, 4, 7); M, 1 kb DNA ladder.

Genomic DNA sequence alignment for *S. aureus* revealed that the sequences of *sa1857*, *yeaZ* (*sa1856*), *sa1855*, and *gcp* (*sa1854*) are sequentially overlapped (Fig. 1A). It was revealed that the first 47 bps of *yeaZ* overlap with the last 47 bps of *sa1857*; the first 28 bps of *sa1855* overlap with the last 28 bps of *yeaZ*; and the first 8 bps of *gcp* overlap with the last 8 bps of *sa1855*. This data suggests that the four genes might be co-transcribed from a common promoter. To examine this possibility, we performed RT-PCR using the forward primers P1for, P2for, and P3for specifically binding to *sa1857*, *yeaZ* and *sa1855*, respectively, and a common reverse primer P4rev specifically binding to *gcp*. The RT negative controls using total RNA as a template did not yielded a PCR product (Fig. 1B, lane 2, 5, 8). In contrast, when cDNA was used as a template, PCR products of 1.4 kb, 800 bp, and 400 bp were obtained, corresponding to the expected sizes (Fig. 1B), indicating that the genes are co-transcribed from at lease one common promoter.

### Staphylococcal Gcp interacts with YeaZ

**Figure 2:**
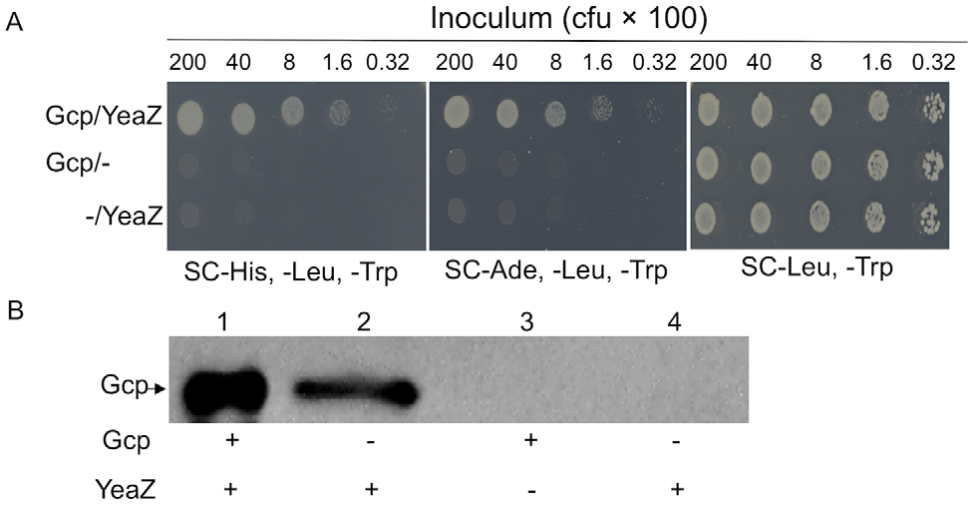
The determination of Gcp binding to YeaZ. (A) Yeast two hybrid analysis of interaction between Gcp and YeaZ on histidine, leucine, and tryptophan drop out synthetic complete (SC-His, -Leu, -Trp) plates with 3-amino-1,2,4,-triazole (3-AT) and SC plates lacking adenine, leucine, and tryptophan (SC-Ade, -Leu, -Trp). Gcp was fused with the activation domain and YeaZ was fused to binding domain. The minus signs indicate empty vector controls. (B) *In vitro* immunoprecipitation analysis of interaction between Gcp and YeaZ. His-tagged Gcp and GST-tagged YeaZ were purified from *E. coli*. Gcp and YeaZ were incubated with Glutathione Sepharose 4B beads together or separately. Western blotting was carried out with rabbit-anti-Gcp serum to detect Gcp. Lane 1: purified Gcp protein was loaded as a positive control.

In *E. coli* the Gcp homolog, YgjD, interacts with YeaZ [25], [26]; in *S. aureus*, *sa1857*, *yeaZ*, *sa1855*, and *gcp* genes are localized to the same operon. These led us to hypothesize that in *S. aureus* Gcp may interact with YeaZ, SA1855, and/or SA1857 and function coordinately. To test this hypothesis, we utilized a yeast two-hybrid system by fusing Gcp, YeaZ, SA1855 and SA1857 separately with the GAL4 activation domain (GAD) and the GAL4 DNA binding domain (GBD), respectively. Two different fusion plasmids were co-transformed into the yeast PJ69-4A competent cells. The results showed that the negative controls carrying pGAD-gcp/pGBD, or pGAD/pGBD empty vectors did not grow in the media lacking His/Leu/Trp, and media lacking Ade/Leu/Trp (Fig. 2A). In contrast, yeast cells carrying pGAD-gcp/pGBD-yeaZ grew normally on the above selective media, indicating a possible binding interaction between Gcp and YeaZ (Fig. 2A). No other interactions were revealed among these four proteins (data not shown). In addition, yeast cells carrying either pGAD-yeaZ/pGBD-gcp or pGAD/pGBD-gcp grew in the above selective media, suggesting that GBD-Gcp fusion may interact with the activation domain leading to an auto-activation of the reporter (data not shown).

To further confirm the interaction between staphylococcal Gcp and YeaZ, we performed *in vitro* pull down assays using the purified His-tagged Gcp and GST-YeaZ fusion proteins. The GST tag was used to immobilize YeaZ on glutathione Sepharose resin. Western blot analysis showed that the YeaZ bound resin was able to retain the refolded Gcp, whereas the control resin alone could not retain the Gcp (Fig. 2B), indicating that the recombinant staphylococcal Gcp specifically binds to the recombinant staphylococcal YeaZ.

The overexpression of Gcp in *E. coli* resulted in the recombinant protein forming inclusion bodies, thus the recombinant protein was purified under denaturing conditions, and refolded by a series of dialysis. Sequence analysis of Gcp revealed four cysteine residues in the C-terminus, thus it is possible for the purified recombinant Gcp molecules to form intermolecular disulfide bonds, which would result in malfunctioning Gcp polymers. To rule out this possibility, we examined the integrity of unfolded and refolded recombinant Gcp proteins by SDS-PAGE under non-reducing condition and did not reveal any oligomers of Gcp (data not shown).

### C-terminal Y317–Y336 segment is crucial for the staphylococcal Gcp binding to YeaZ

**Figure 3:**
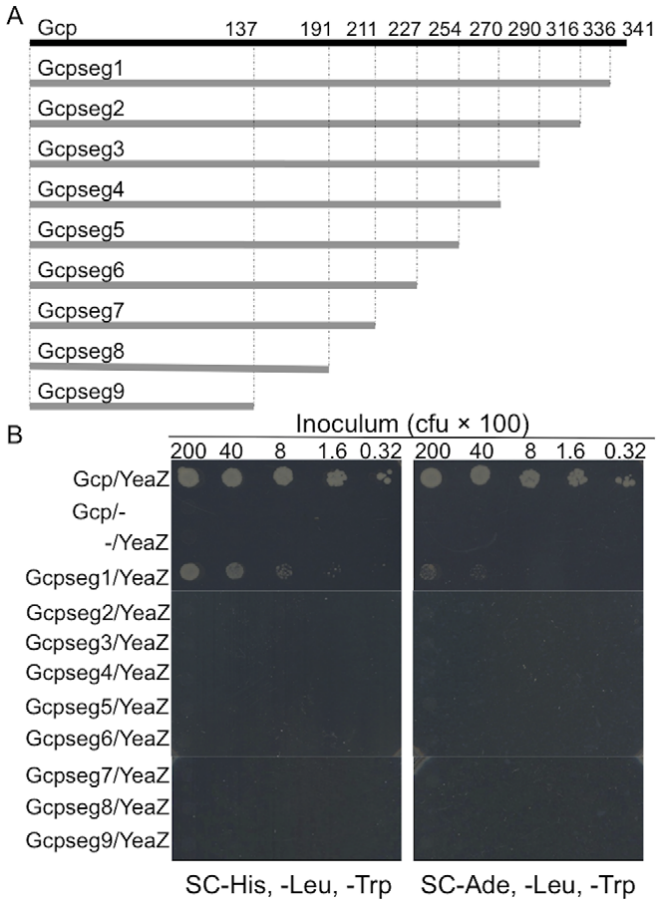
Diagrams represent different deletion mutations of Gcp (A); and interactions between *S. aureus* Gcp and YeaZ detected by yeast two-hybrid analyses (B). Yeast cells containing the indicated plasmids were serially diluted, and 5 µl of diluted cells was spotted on the plate with media lacking histidine, leucine, and tryptophan; or lacking adenine, leucine, and tryptophan to score for interactions after incubation at 30°C.

In order to identify which domains of staphylococcal Gcp are important for the Gcp-YeaZ interaction, we created nine different truncated Gcp mutants by PCR (Fig. 3A). These truncated Gcp segments were fused with the activation domain of pGAD, respectively, and the resulting recombinant plasmids were utilized to conduct Y2H analyses with pGBD-yeaZ. The results showed that the yeast cells carrying Gcpseg1 mutant (E337–E341 truncated Gcp) and YeaZ grew on both SC-His, -Leu, -Trp and SC-Ade, Leu, -Trp selective plates. However, the yeast cells carrying other truncated Gcp fusions and YeaZ did not grow on these selective plates (Fig. 3B).

**Figure 4:**
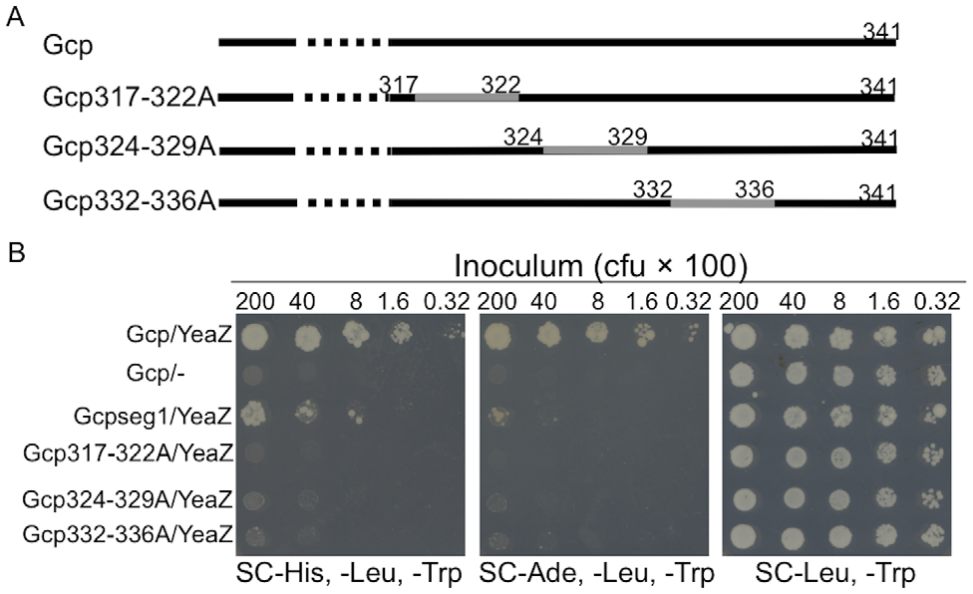
Diagrams represent different alanine substitutions of amino acids in Gcp (A); and interactions between *S. aureus* Gcp and YeaZ detected by yeast two-hybrid analyses (B). Yeast cells containing the indicated plasmids were serially diluted, and 5 µl of diluted cells was spotted on the plate with media lacking histidine, leucine, and tryptophan (SC-His, -Leu, -Trp); or lacking adenine, leucine, and tryptophan (SC-Ade, -Leu, -Trp) to score for interactions after incubation at 30°C.

Alanine scanning mutagenesis has been successfully and extensively utilized in the determination of enzymatic and functional residues of proteins [30]–[32]. Substitution of alanine removes all side chain atoms beyond β carbon and yet maintains the main chain structure [30]. To further identify which residue(s) is required for Gcp binding to YeaZ, we utilized this approach to substitute potential hot residues of the C-terminal Y317–Y336 segment with alanine. The critical Y317–Y336 segment was divided into three regions, including Y317-F322, D324-N329, and S332-Y336 regions as shown in Fig. 4A. Five to six amino acids in these regions were replaced by alanine stretches, respectively. The mutated *gcp* fragments were fused with the activation domain of pGAD, which was used for the Y2H assays. We found that the yeast cells carrying with Gcp317-322A and YeaZ did not grow on any SC dropout selective medium (Fig. 4B); the yeast cells carrying Gcp324-329A or Gcp332-336A, and YeaZ appeared to have a dramatic growth defect on the selective medium (Fig. 4B). Taken together, the above data indicate the importance of the C-terminal region of Gcp for proper interaction with YeaZ, specifically the region of Y317-F322 and to a lesser extent the regions of D324-N329 and S332-Y336.

### Staphylococcal Gcp alanine mutants cannot complement wild type Gcp for bacterial growth

**Figure 5:**
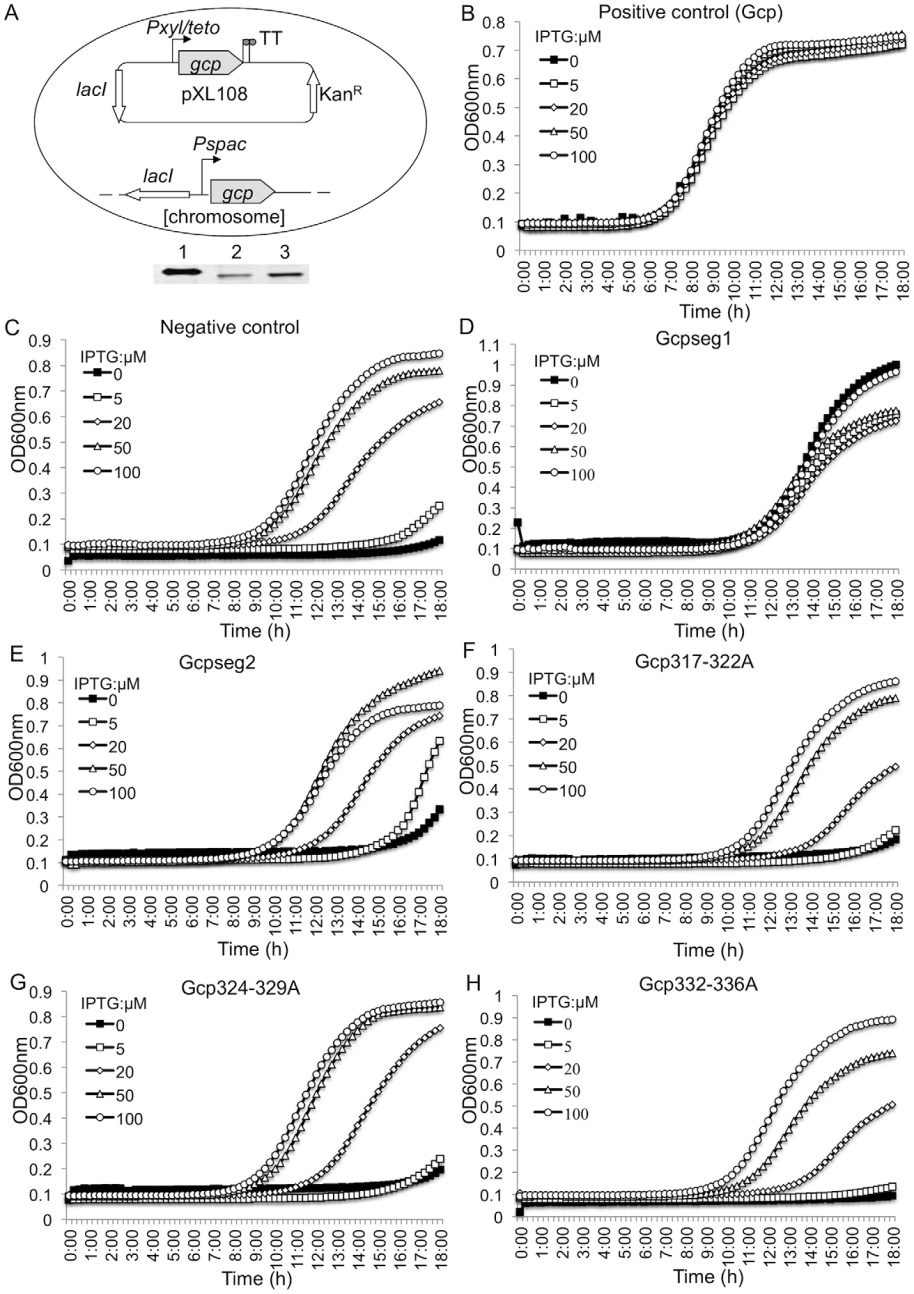
The impact of the expression of wild type or mutated Gcp in trans on the growth of the Pspac-regulated gcp expression strain. (A) Construction of Gcp complementation system using the *Pspac*-regulated *gcp* expression strain and Western blot analysis of *gcp* expression *in trans*. Lane 1, purified recombinant Gcp; lane 2, negative control (JRN0210); lane 3, Gcp complementary strain (JNR0110). Without an inducer, ATc, Gcp is constitutively expressed due to the leakage of *Pxyl/tetO* promoter. (B–H) Growth curves of Gcp complementary strains. The *Pspac*-regulated *gcp* expression *S. aureus* strain carrying the staphylococcal wild-type *gcp* complementary plasmid, pXL108 was used as positive control (B); carrying parental plasmid, pMY1107, as a negative control (C); carrying the *gcpseg1* complementary plasmid, pLT109 (D); carrying the *gcpseg2* complementary plasmid, pLT209 (E); carrying the *gcp1-1* complementary plasmid, pLT309 (F); carrying the *gcp1-2* complementary plasmid, pLT409 (G); carrying the *gcp1-3* complementary plasmid, pLT509 (H). The above strains were incubated overnight in TSB in the presence of appropriate antibiotics and 0.2 mM IPTG. Bacteria were diluted and incubated in fresh TSB containing appropriate antibiotics and different concentration of IPTG at 37°C with shaking. The cell growth was monitored by measuring OD_600 nm_ every 15 min for 18 h with 1 min mixing before each reading.

We have previously demonstrated that Gcp is essential for *S. aureus* growth [5], [6], but the molecular mechanisms of Gcp's essentiality remain elusive. The discovery that Gcp interacts with YeaZ led us to hypothesize that their interaction is important for Gcp's essentiality. In order to examine this hypothesis, we first created the Gcp complementary system (Fig. 5A). Then, we examined the complementary effect of *gcp* expression *in trans* by measuring the bacterial growth during the depletion of endogenous Gcp. The results showed that the growth of the staphylococcal Gcp complementary strain was IPTG-independent, indicating the *gcp* expression *in trans* complements the depletion of endogenous Gcp (Fig. 5B), whereas the growth of the parental control was IPTG-dependent (Fig. 5C). These data further demonstrated the requirement of Gcp for bacterial viability in culture and the feasibility of the Gcp complementary system.

Next, we determined whether the identified segment and regions that are crucial for Gcp interaction with YeaZ are also required for growth by examining the complementary effect of the mutated Gcp genes. The results showed that the growth of the *Pspac*-regulated *gcp* expression strain carrying complementary Gcpseg1 was independent of IPTG (Fig. 5D), indicating that the expression of Gcpseg1 *in trans* can complement Gcp and the C-terminal region E337–E341 is dispensable for Gcp's essentiality. In contrast, the growth of the *Pspac*-regulated *gcp* expression strain carrying complementary Gcpseg2 was still dependent on IPTG (Fig. 5E). To determine which domain is critical for growth, we examined the complementary effect of Gcp317-322A, Gcp324-329A and Gcp332-336A, and found that the *Pspac*-regulated *gcp* expression strains carrying Gcp317-322A (Fig. 5F), Gcp324-329A (Fig. 5G), or Gcp332-336A (Fig. 5H) mutant exhibited IPTG dependent growth, indicating that these mutated Gcp are unable to complement the depletion of endogenous Gcp. These data suggest that the three domains, Y317-F322, D324-N329, and S332-Y336, are likely critical for Gcp's essentiality.

### No *O*-sialoglycoprotein endopeptidase activity was detected for the recombinant staphylococcal Gcp and YeaZ proteins

**Figure 6:**
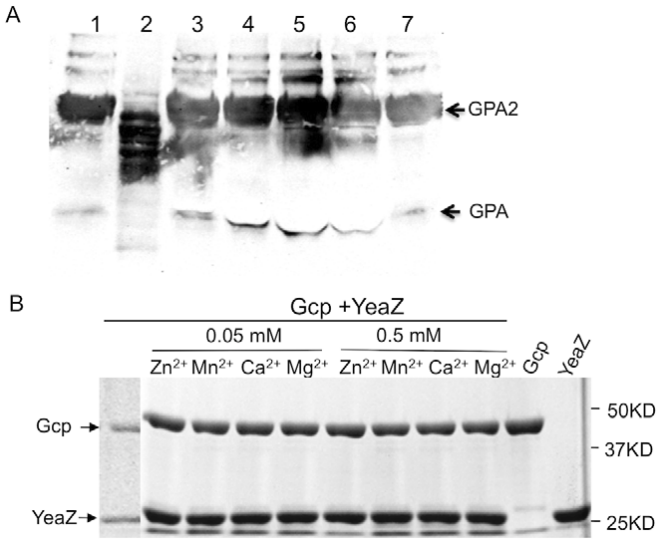
SDS-PAGE and Western blot analysis of Gcp and YeaZ's protease activity. (A) Western blot results showing hydrolysis of Glycophorin A. GPA2 and GPA represent dimer and monomer of glycophorin A. 10 µg glycophorin A was incubated with PBS (lane 1), 5 µg *O*-sialoglycoprotein endopeptidase (Gcp) of *P. haemolytica* (Cedarlane Laboratories, lane 2), 5 µg Gcp (lane 3), 25 µg Gcp (lane 4), 50 µg Gcp (lane 5), 33 µg YeaZ (lane 6) and 5 µg Gcp and 3.3 µg YeaZ (lane 7). (B) SDS-PAGE analysis of the impact of the recombinant staphylococcal YeaZ on Gcp.

To determine whether the novel essential proteins Gcp and YeaZ possess an *O*-sialoglycoprotein endopeptidase activity, the purified recombinant Gcp and YeaZ were utilized to treat *O*-sialoglycoprotein, glycophorin A, according to the manufacturer's instruction. The degradation of glycophorin A protein was detected by SDS-PAGE and western blot using a monoclonal antibody against glycophorin A. Significant degradation of glycophorin A was detected after treatment with Gcp of *M. haemolytica* A1 (Fig. 6A), whereas no degradation of glycophorin A was detected after treatment with recombinant Gcp, YeaZ, or the combination of Gcp and YeaZ (Fig. 6A). In addition, we revealed that the purified recombinant staphylococcal YeaZ did not cleave recombinant Gcp of *S. aureus* under the conditions in the presence or absence of Mg^2+^, Mn^2+^, Ca^2+^, Zn^2+^ (Fig. 6B).

## Discussion

In the present study, we employed genetic and biochemical approaches and demonstrated that in the *gcp* operon of *S. aureus*, the essential Gcp protein interacts with another essential protein YeaZ. More importantly, we identified key domains of Gcp that are not only required for Gcp binding to YeaZ, but also play important roles in Gcp's essentiality for bacterial growth. Our results suggest that the interaction of Gcp and YeaZ may contribute to the essentiality of Gcp. In addition, our results showed that there was neither *O*-sialoglycoprotein endopeptidase activity nor Gcp specific protease activity detected for the purified recombinant staphylococcal Gcp and/or YeaZ, suggesting that the staphylococcal Gcp and YeaZ may function differently compared to their homologs in *M. hemolytica* and *E. coli*, respectively. These findings may provide new insights into the molecular mechanisms and biological function of the essential protein, Gcp.

The identification of the staphylococcal Gcp-YeaZ interaction is consistent with the previous report that the Gcp homolog in *E. coli*, YgjD, binds to YeaZ the homolog of SA1856 [25], [26]. However, in *E. coli* another essential protein, YjeE, also interacts with YeaZ, suggesting that YjeE may function as a regulator to modulate YeaZ-YgjD interaction, and that the YjeE-YeaZ-YgjD network may be involved in an essential cellular process [26]. In contrast, in *S. aureus* there is no report indicating that the homolog of YjeE, SA1857, is indispensable for bacterial viability [10]. We are in the process of examining the requirement of SA1857 and SA1855 for the growth of *S. aureus*. Furthermore, in our Y2H studies we found no evidence that SA1857 and SA1855 interacts with either Gcp or YeaZ. Although the staphylococcal YeaZ and Gcp proteins have approximately 26% and 42% identity compared to *E. coli* YeaZ and YgjD sequences, respectively, our studies indicate that Gcp and YeaZ likely function differently or interact with different partner proteins compared to their homologs in *E. coli*. We observed that in Y2H assays the growth of yeast cells on SC-His plates seems to be faster than on SC-Ade plates. This is likely due to the stringent growth difference between SC-Ade and SC-His selective medium [33]. Although we unveiled that the mutation of Y317-F322 region eliminated the ability of Gcp to bind YeaZ, and the mutations of D324-N329 and S332-Y336 regions alleviated the capacity of Gcp to bind YeaZ, the attenuated binding ability may be attributable to the altered tertiary conformation of Gcp. To determine the potential impact of topology for mutated Gcp proteins is beyond scope of the present study. However, we are currently working to further characterize and define the interaction of Gcp and YeaZ and identify the critical residue(s) for Gcp to bind YeaZ and vice versa.

In both yeast and archaea, Kae1p (Gcp homolog) directly interacts with a Bud32p kinase, inhibiting the kinase activity, which is required both for transcription and the telomere homeostatic function of the endopeptidase-like kinase chromatin-associated (EKC)/kinase, endopeptidase, and other peptidases of small size (KEOPS) in yeast cells [21], [23], [24]. In eukaryotes, the Gcp homolog is Qri7 in yeast and OSGEPL1 (*O*-sialoglycoprotein endopeptidase) in worm and human. Qri7 and OSGEPL1 anchor to the mitochondrial inner membrane and are essential for the maintenance of mitochondrial genome [18], [34]. Qri7 is able to complement the depletion of *E. coli* YgjD (homolog of Gcp), whereas Kae1 fails to do so, suggesting the functional similarity between Qri7 and YgjD [34]. Although bacteria lack a Bud32 homolog, YeaZ may substitute for Bud32 to form a functional complex with Gcp. Our complementation experiments established that the mutations of Gcp that disrupt the Gcp-YeaZ interaction could not complement the depletion of endogenous Gcp. We also observed that the deletion of the C-terminal residues, E337–E341, slightly impaired the ability of Gcp to interact with YeaZ (Fig. 3B); and consequently, this truncated Gcp (Gcpseg1) delayed its complementary effect on bacterial growth (longer lag-phase of growth). These data suggest that the staphylococcal Gcp-YeaZ interaction may play an important role in Gcp's essentiality; thus the interruption of Gcp-YeaZ interaction may be utilized as a novel mode of action for developing new classes antibacterial agents, partially against MRSA or VRSA caused infections. It is known that bacterial autolysis is important for bacterial cell division and growth. Previously, we have demonstrated that the staphylococcal Gcp is a critical modulator of bacterial autolysis [6]; however, the mechanism of regulation of autolysis by Gcp remains to be determined; it is necessary to explore the potential role of the Gcp-YeaZ interaction in the process of autolysis.

Our purified recombinant staphylococcal Gcp and YeaZ did not appear to have an *O*-sialoglycoprotein endopeptidase activity against glycophorin A. We also employed an alternative approach to examine the potential glycoprotease activity by measuring cell associated ligand, PSGL1 (P-selectin glycoprotein ligand 1), using FACS. Neither the purified recombinant staphylococcal Gcp, YeaZ, nor the concentrated supernatants of *S. aureus* culture appeared to have the glycoprotease activity (data not shown). Our results are in agreement with previous reports that YgjD (homolog of Gcp in *E. coli*) and Kae1 (homolog of Gcp in *Pyrococcus abyssi*) do not exhibit any endopeptidase activity [20], [26]. In addition, E. coli YeaZ is able to specifically cleave YgjD (the homolog of Gcp), whereas consistent with *S. typhimurium* YeaZ [28] our purified soluble recombinant staphylococcal YeaZ did not exhibit such a protease activity. The lack of protease activity of purified recombinant Gcp and YeaZ may result from potential modifications during expression and purification process. However, it is likely that the Gcp and YeaZ homologs may have different biological functions among different species, because sequence alignment analysis revealed that the staphylococcal Gcp does not possess any *E. coli* YeaZ cleavage sites, including K171- L172 and F195-V196 residues that were identified in YgjD [26]. In addition, we cannot exclude the possibility that the staphylococcal Gcp and YeaZ proteins may possess a specific proteolytic activity against a substrate in a process that is critical for the viability of *S. aureus*.

In conclusion, we demonstrated within the four novel proteins encoded by the gcp operon, the essential Gcp interacts with another essential protein YeaZ of *S. aureus*. Moreover, we identified the C-terminal Y317-F322, D324-N329, and S332-Y336 regions to be important for Gcp to bind YeaZ, as well as for Gcp's essentiality, whereas the C-terminal E337–E344 region is dispensable for the staphylococcal Gcp-YeaZ interaction and Gcp's essentiality. These data suggest that the interaction of Gcp and YeaZ may at least partially contribute to the essentiality of Gcp for *S. aureus* growth. Our findings provide new insights into the potential mechanisms and biological function of the novel essential protein, Gcp, as well as potential novel targets for the development of new classes antibacterial agents.

## Materials and Methods

### Bacterial strain and culture medium

The bacterial strains and plasmids used in the study are listed in Table 1. *E. coli* strain, DH10B, was used as a host for recombinant plasmid construction and amplification. Luria-Bertani (LB) liquid medium and LB-agar plates were used for the growth and maintenance of *E. coli*. The *E. coli* strain BL21(DE3) was used as a host strain for the expression of recombinant Gcp and YeaZ. Ampicillin was used at 100 µg/ml in LB media for the selection of *E. coli* carrying the plasmid pGEX4T-1, pMY1107, or Yeast two-hybrid (Y2H) vectors pGAD-C1 and pGBD-C1. Kanamycin was used at 50 µg/ml in LB media for the selection of *E. coli* carrying plasmid pET24b. A *Saccharomyces cerevisiae* strain, PJ69-4A, was used in Y2H studies [33]. The PJ69-4A strain has three reporter genes for a positive Y2H interaction: *HIS3* driven by *GAL1* promoter, *ADE2* by the *GAL2* promoter, and *lacZ* by the *GAL7* promoter. YEPD (1% yeast extract, 2% peptone, 2% dextrose, pH 6.0) and synthetic complete (SC) liquid medium and plates were used for *S. cerevisiae* growth. Where indicated, certain components were dropped out from the SC media. A *Pspac*-regulated *gcp* mutant of *S. aureus* was constructed as previously described [5], which was used as a host cell for complementation experiments. *S. aureus* was grown in Tryptic Soy Broth (TSB) containing erythromycin (5 µg/ml) and different concentrations of isopropyl β-D-1-thiogalactopyranoside (IPTG) at 37°C with shaking.

**Table 1:** Strains and plasmids used in this study.

Strain or plasmid	Description	Reference
Strains		
RN4220	Laboratory strain; *rsbU^−^*	[39]
WCUH29	Clinical human isolate; *rsbU* ^+^	[40]
JRN0105	RN4220::*Pspac*-*gcp*, *spac* promoter regulated *gcp* expression mutant	[5]
JRN0110	JRN0105 with plasmid pXL108	This study
JRN0210	JRN0105 with plasmid pMY1107	This study
JRN0310	JRN0105 with plasmid pLT109	This study
JRN0410	JRN0105 with plasmid pLT209	This study
JRN0510	JRN0105 with plasmid pLT309	This study
JRN0610	JRN0105 with plasmid pLT409	This study
JRN0710	JRN0105 with plasmid pLT509	This study
PJ69-4A	*MATa trp1-901 leu2-3, 112 ura3-52 his3-200 gal4Δgal80ΔLYS2::GAL1-HIS3 GAL2-ADE2 met2::GAL7-lacZ*	[33]
Plasmids		
pGEX4T-1	Overexpression vector, can be used to add GST tag to the N terminal of protein of interest	GE Healthcare
pET-24b	Overexpression vector, can be used to add His tag to the N or C terminal of protein of interest	Novagen
pGAD-C1/pGBD-C1	Y2H vector carrying *GAL4* transcription activation/DNA binding domain	[33]
pGAD/gcp	pGAD-C1 carrying *gcp*	This study
pGAD/gcpseg1	pGAD-C1 carrying *gcpseg1* mutant	This study
pGAD/gcpseg2	pGAD-C1 carrying *gcpseg2* mutant	This study
pGAD/gcpseg3	pGAD-C1 carrying *gcpseg3* mutant	This study
pGAD/gcpseg4	pGAD-C1 carrying *gcpseg4* mutant	This study
pGAD/gcpseg5	pGAD-C1 carrying *gcpseg5* mutant	This study
pGAD/gcpseg6	pGAD-C1 carrying *gcpseg6* mutant	This study
pGAD/gcpseg7	pGAD-C1 carrying *gcpseg7* mutant	This study
pGAD/gcpseg8	pGAD-C1 carrying *gcpseg8* mutant	This study
pGAD/gcpseg9	pGAD-C1 carrying *gcpseg9* mutant	This study
pGAD/gcp1-1	pGAD-C1 carrying *gcp1-1* mutant	This study
pGAD/gcp1-2	pGAD-C1 carrying *gcp1-2* mutant	This study
pGAD/gcp1-3	pGAD-C1 carrying *gcp1-3* mutant	This study
pGAD/sa1855	pGAD-C1 carrying *sa1855*	This study
pGAD/yeaZ	pGAD-C1 carrying *yeaZ*	This study
pGAD/sa1857	pGAD-C1 carrying *sa1857*	This study
pGBD/gcp	pGBD-C1 carrying *gcp*	This study
pGBD/sa1855	pGBD-C1 carrying *sa1855*	This study
pGBD/yeaZ	pGBD-C1 carrying *yeaZ*	This study
pGBD/sa1857	pGBD-C1 carrying *sa1857*	This study
pMY1107	pFF40 inserted with TetR regulation region	This study
pXL108	pMY1107 carrying *gcp*	This study
pLT109	pMY1107 carrying *gcpseg1* mutant	This study
pLT209	pMY1107 carrying *gcpseg2* mutant	This study
pLT309	pMY1107 carrying *gcp1-1* mutant	This study
pLT409	pMY1107 carrying *gcp1-2* mutant	This study
pLT509	pMY1107 carrying *gcp1-3* mutant	This study

### RNA purification and RT-PCR analysis

Overnight cultures of *S. aureus* were used at 1% inoculum in TSB medium and grown to the medium-exponential phase of growth. Cells were harvested by centrifugation, and the total RNA was isolated by using the RNAPrep kit (Promega, MI). Contaminating DNA was removed with a TURBO DNA-*free* Kit (Ambion), and the RNA yield was determined spectrophotometrically at 260 nm. The cDNA was synthesized by employing RNA as a template using the Promega-reverse transcription kit. Co-transcription of *gcp* operon was determined by RT-PCR using primers listed in Table 2.

**Table 2:** Primers used in this study.

Name	Sequence
P1for	CTGATGAAGATTAGGGTTTGATGA
P2for	TTACAAGATGAATTACAAGGTGAAGTGA
P3for	TTATCGATCAAGCTCAAATTACAACAG.
P4rev	ACTAGAGCCTCGTTATTTGTTGTTGTTA
gcpforEcoRI	TT**GAATTC**ATGACTAAAGATATATTAATACTAGC
gcprevBglII	TT**AGATCT**TTCTGCAGAATACTCTTCTA
gcpseg1RevBglII	GC**AGATCT**TTATAAATCTATATTGCTGTGCC
gcpseg2RevBglII	TT**AGATCT**TTACAAAGAGTGGCCGGCA
gcpseg3RevBglII	TT**AGATCT**TTAATTGACTTTGCATTGATCC
gcpseg4RevBglII	TT**AGATC**TTTAAGCAACAATTAATCGCTGA
gcpseg5RevBglII	TT**AGATCT**TTATTTAAACGTAAGCACCTCTAC
gcpseg6RevBglII	TT**AGATCT**TTATTGATTGTGAAGTTGATTGAT
gcpseg7RevBglII	TT**AGATCT**TTAAAAATCATAACTATCTTTATCCAA
gcpseg8RevBglII	TT**AGATCT**TTAAGCAGCCAACCGATCAA
gcpseg9RevBglII	TT**AGATCT**TTAAATAAGTGCAATTAGCGGG
gcpfor1	AAACTATGACTAAAGATATATTAATACTAG
gcprevAscI	TT**GGCGCGCC**TTCTGCAGAATACTCTTCTA
gcpseg1revAscI	TT**GGCGCGCC**TTATAAATCTATATTGCTGTGCC
gcpseg2revAscI	TT**GGCGCGCC**TTACAAAGAGTGGCCGGCA
gcp1-1forSacII	GCTG**CCGCGG**CTGCTGCTGCTGATTTAGCATTAAAT
gcp1-1revSacII	AGCAGCAG**CCGCGG**CAGCCAAAGAGTGGCCGGCAAC
gcp1-2forSacII	GCTG**CCGCGG**CTGCTGGGCACAGCAATATAGAT
gcp1-2revSacII	AGCAG**CCGCGG**CAGCAGCAAATCGACCTTGCTG
gcp1-3forSacII	GCTG**CCGCGG**CTGCTGAAGAGTATTCTGCAGAA
gcp1-3revSacII	AGCAG**CCGCGG**CAGCGTGCCCATTTAATGCTAA
gcpmutrevAscI	TT**GGCGCGCC**TTATTCTGCAGAATACTCTTC
gcppETforNdeI	GGAATTC**CATATG**ACTAAAGATATATTAATACTAG
gcppETrev	CCGCTCGAGTTCTGCAGAATACTCTTCTA
yeaZforBamHI	TCGC**GGATCC**ATGAACAAATTAAGGAGGCAAT
yeaZrevSalI	CTCC**GTCGAC**TTAATTGTTCTTTTGACTGTTGA
sa1857BamHI	TCGC**GGATCC**TTGATAAAGATAAATAATTTAGATG
sa1857SalI	CTCC**GTCGAC**TCAATGAGCAGCGAATTCATG
sa1855BamHI	TCGC**GGATCC**TTGGATCAACAGTCAAAAGAAC
sa1855SalI	CTCC**GTCGAC**TTAGTCATTTAAATTCACCCAC

### Examination of protein interactions using the Y2H system

To examine possible protein-protein interaction, we employed a yeast two-hybrid (Y2H) technology as described [33], [35]. The staphylococcal *gcp*, *sa1855*, *yeaZ* and *sa1857* genes were amplified by PCR using the primers listed in Table 1. PCR products were purified, cloned into *EcoR*I and *Bgl*II sites of pGAD-C1 two-hybrid vector containing GAL4 transcription activation domain (GAL4AD) and *Bam*HI and *Sal*I sites of pGBD-C1 vector containing GAL4 DNA-binding domain (GAL4BD), respectively. The yeast strain PJ69-4A was co-transformed with the two fusion plasmids using the high efficiency LiAc-PEG method [35]. The yeast transformants were grown on SC drop-out plates lacking leucine and tryptophan at 30°C. The examination was performed either by streak plates or by spotting plates with serial dilutions of suspension on SC plates lacking leucine, tryptophan and histidine with 2 mM 3-amino-1,2,4,-triazole (3-AT) (SC-His, -Leu, -Trp) and SC plates lacking leucine, tryptophan and adenine (SC-Ade, -Leu, -Trp). For the spots assays, yeast cells in exponential growth were harvested and resuspended at 4×10^6^ cells per milliliter. A volume of 5 µl of this cell suspension and its 5 fold serial dilutions were spotted on plates with decreasing concentrations from left to right. Growth on plates was examined daily and documented after 4 days of incubation.

### Cloning, expression and purification of recombinant Gcp-His tag fusion protein and GST-YeaZ fusion protiens

The staphylococcal *gcp* and *yeaZ* genes were obtained by PCR using the primers listed in Table 2 and cloned into *Nde*I and *Xho*I sites of pET24b and *Bam*HI and *Sal*I sites of pGEX4T-1, respectively. The recombinant plasmids were transformed into *E. coli* strain BL21 (DE3), respectively. An approximately 500 ml culture of each strain in LB medium was incubated aerobically at 37°C to an optical density (OD) at 600 nm of 0.6. The recombinant protein expression was subsequently induced by the addition of 1 mM IPTG. After 5 h of incubation at 37°C, cells were harvested. For the purification of Gcp-His fusion proteins, the cell pellet was collected and resuspended in the binding buffer (300 mM NaCl, 50 mM NaH_2_PO_4_, 8 M urea, 10 mM imidazole, pH 8.0) with the addition of 1 mg/ml lysozyme and 1 mM PMSF at room temperature for 1 h. The cells were lysed by sonication on ice; after centrifugation at 25,000 g for 20 min at 4°C, the supernatant was collected and applied onto Ni-NTA resin (Qiagen) under denaturing condition according to the manufacturer's instruction. The purified recombinant Gcp proteins were refolded by dialysis using a series of dialysis buffers (300 mM NaCl, 50 mM NaH_2_PO_4_, 10% glycerol with 4 M, 2 M, 1 M, 0 M urea, final buffer 150 mM NaCl, 20 mM NaH_2_PO_4_, PH 7.3). For the purification of GST-YeaZ proteins, the cell pellet was resuspended in the binding buffer (150 mM NaCl, 20 mM NaH_2_PO_4_, pH 7.3) containing 1 mg/ml lysozyme and 1 mM PMSF at room temperature for 1 h. The cells were lysed by sonication on ice; after centrifugation at 25,000 g for 20 min at 4°C, the supernatant was collected and mixed with Glutathione Sepharose 4B beads (GE Healthcare) according to the manufacturer's instructions. The purified recombinant proteins were confirmed by SDS-polyacrylamide gel electrophoresis (PAGE) followed by Coomassie bright blue staining; the protein concentration was measured using the BCA™ Protein Assay kit (Thermo Scientific).

### *In vitro* pull down assay

Purified GST-YeaZ fusion protein was mixed with Glutathione Sepharose 4B resin at room temperature and allowed to bind for 30 min. A total of 15 µl of resin bound GST-YeaZ protein was incubated with the molar equivalents of purified Gcp-His tag protein for 2 h at room temperature, washed 6 times with phosphate buffer solution using the equivalent volume of 100 times the bead bed volume, and eluted by mixing with 2× the bead bed volume of elution buffer containing 10 mM reduced glutathione. The elution fraction was subjected to SDS-PAGE and standard western blotting using rabbit anti-Gcp serum.

### Alanine scanning mutagenesis

To identify the domains of Gcp important for binding to YeaZ, we performed alanine scanning mutagenesis assays. Five to six amino acids of alanine per stretch replaced the native amino acids and were generated by PCR using the primers listed in Table 2 as previously described [36]. In brief, six pairs of primers were designed, including Gcpfor1 with Gcp1-1revSacII, Gcp1-2revSacII and Gcp1-3revSacII, and GcprevAscI with Gcp1-1forSacII, Gcp1-2forSacII and Gcp1-3forSacII (Table 2). Three different 5′ end segments and 3′ end segments of *gcp* were amplified by PCR, ligated to *Sac*II site, and reformed mutated *gcp1-1*, *gcp1-2*, and *gcp1-3*, respectively. These mutated genes encode three different C-terminal alanine stretch mutants, Gcp317-322A, Gcp324-329A, and Gcp332-336A, accordingly.

### Construction of complementary plasmid and characterization of bacteria growth

In order to examine whether the expression of wild type or the constructed alanine mutants of Gcp *in trans* are able to complement the depletion of endogenous Gcp, we created a complementary plasmid using a shuttle vector, pFF40, which expresses the *lacI* gene [37]. A TetR regulatory cassette, obtained by PCR, was inserted into the *Sac*II site of pFF40 and the new plasmid was designated as pMY1107. The wild type and alanine mutant *gcp* genes were obtained by PCR using the primers listed in Table 2 and cloned into the *Pme*I and *Asc*I sites of pMY1107, resulting in plasmids pXL108, pLT109, pLT209, pLT309, pLT409, and pLT509. The recombinant plasmids were electrophorated into the *Pspac*-regulated *gcp S. aureus* mutant [5]. The growth curves of transformed *S. arueus* were obtained using an automated microtiter plate format on SpectrMaxPlus384 spectrophotometer (Molecular Devices). *S. aureus* strains were incubated in TSB with appropriate antibiotics and different concentrations of IPTG (0, 5, 20, 50 and 100 µM) at 37°C. Cell growth was monitored by measuring OD_600 nm_ every 15 min with 1 min mixing before each reading.

### Enzymatic activity analysis

To determine whether the staphylococcal Gcp and YeaZ possess any *O*-sialoglycoprotein endopeptidase activity or protease activity, purified recombinant Gcp and YeaZ proteins were utilized to treat *O*-sialoglycoprotein glycophorin A (Sigma) as described [38]. The degradation of glycophorin A protein was detected by SDS-PAGE and western blotting using a monoclonal antibody against glycophorin A. To examine the effect of different ions, including magnesium, calcium, and zinc on the potential enzymatic activity, the purified recombinant Gcp and/or YeaZ was incubated with glycophorin A in individual reaction buffers containing different concentrations of Mg^2+^, Mn^2+^, Ca^2+^, or Zn^2+^ at 37°C for 3 h.
